# Modelling neurofibromatosis type 1 tibial dysplasia and its treatment with lovastatin

**DOI:** 10.1186/1741-7015-6-21

**Published:** 2008-07-31

**Authors:** Mateusz Kolanczyk, Jirko Kühnisch, Nadine Kossler, Monika Osswald, Sabine Stumpp, Boris Thurisch, Uwe Kornak, Stefan Mundlos

**Affiliations:** 1 Max Planck Institute for Molecular Genetics, FG Development & Disease, Berlin, Germany; 2 Institute for Medical Genetics, Charité, Universitätsmedizin Berlin, Berlin, Germany; 3 In Silico Miners, ul. Chopina 13/10, 81-782 Sopot, Poland; 4 Berlin-Brandenburg Center for Regenerative Therapies (BCRT), Berlin, Germany

## Abstract

**Background:**

Bowing and/or pseudarthrosis of the tibia is a known severe complication of neurofibromatosis type 1 (NF1). Mice with conditionally inactivated neurofibromin (Nf1) in the developing limbs and cranium (Nf1Prx1) show bowing of the tibia caused by decreased bone mineralisation and increased bone vascularisation. However, in contrast to NF1 patients, spontaneous fractures do not occur in Nf1Prx1 mice probably due to the relatively low mechanical load. We studied bone healing in a cortical bone injury model in Nf1Prx1 mice as a model for NF1-associated bone disease. Taking advantage of this experimental model we explore effects of systemically applied lovastatin, a cholesterol-lowering drug, on the Nf1 deficient bone repair.

**Methods:**

Cortical injury was induced bilaterally in the *tuberositas tibiae *in Nf1Prx1 mutant mice and littermate controls according to a method described previously. Paraffin as well as methacrylate sections were analysed from each animal. We divided 24 sex-matched mutant mice into a lovastatin-treated and an untreated group. The lovastatin-treated mice received 0.15 mg activated lovastatin by daily gavage. The bone repair process was analysed at three consecutive time points post injury, using histological methods, micro computed tomography measurements and *in situ *hybridisation. At each experimental time point, three lovastatin-treated mutant mice, three untreated mutant mice and three untreated control mice were analysed. The animal group humanely killed on day 14 post injury was expanded to six treated and six untreated mutant mice as well as six control mice.

**Results:**

Bone injury repair is a complex process, which requires the concerted effort of numerous cell types. It is initiated by an inflammatory response, which stimulates fibroblasts from the surrounding connective tissue to proliferate and fill in the injury site with a provisional extracellular matrix. In parallel, mesenchymal progenitor cells from the periost are recruited into the injury site to become osteoblasts. In Nf1Prx1 mice bone repair is delayed and characterised by the excessive formation and the persistence of fibro-cartilaginous tissue and impaired extracellular matrix mineralisation. Correspondingly, expression of Runx2 is downregulated. High-dose systemic lovastatin treatment restores Runx2 expression and accelerates new bone formation, thus improving cortical bone repair in Nf1Prx1 tibia. The bone anabolic effects correlate with a reduction of the mitogen activated protein kinase pathway hyper-activation in Nf1-deficient cells.

**Conclusion:**

Our data suggest the potential usefulness of lovastatin, a drug approved by the US Food and Drug Administration in 1987 for the treatment of hypercholesteraemia, in the treatment of Nf1-related fracture healing abnormalities. The experimental model presented here constitutes a valuable tool for the pre-clinical stage testing of candidate drugs, targeting Nf1-associated bone dysplasia.

## Background

Long bone pseudarthrosis, usually of the tibia, is a well known and serious complication of neurofibromatosis type 1 (NF1) [1-3]. The condition presents within the first years of life either as bowing of the affected bone, or with an hourglass constriction and subsequent spontaneous fracture. The aetiology of the condition has never been well established, and its exact cause is unknown. Therapeutic programs have been largely based on conceptual considerations for the treatment of post-traumatic non-unions. These forms of treatment, however, are often futile when applied to pseudarthrosis of the tibia indicating that systemic problems interfere with normal healing. In some cases amputation is the only option.

In order to better understand neurofibromin (Nf1) function in bone we recently generated mice bearing a homozygous Nf1 inactivation in the embryonic limb and in the cranial mesenchyme. The affected cell types include endothelial cells, chondrocytes and osteoblasts but not osteoclasts, which are of haematopoietic origin. Interestingly, early limb bud specific Nf1 inactivation results in tibia bowing similar to that observed in NF1 patients [4]. However, since in the mouse model the affected extremities are not subjected to excessive mechanical force, bone fracture and the expected pseudarthrosis never occur spontaneously. In order to study the role of Nf1 in the regulation of bone repair we applied a previously described bone injury model, which has been designed for the comparative analysis of the bone healing in wild-type versus knock-out mice [5,6]. The model involves drilling 0.5 mm holes through the entire diameter of the tibial diaphysis, which does not lead to bone shaft breakage, as the remaining cortical structure stabilises the bone collar. Despite the small size of the injury, the experimental model enables both qualitative and quantitative analysis of the complex process of bone repair. At the same time it causes the least possible distress to the tested animals. The normal repair process involves stages of haematoma formation, connective tissue fibroblast and mesenchymal stem cell recruitment followed by osteoblast differentiation. Consequently, bone formation in the course of the injury repair relies on the timely recruitment and differentiation of mesenchymal progenitor cells within the injury site. These processes appear disturbed in Nf1Prx mice leading to a delay of cortical bone regeneration accompanied by the accumulation of the fibro-cartilaginous tissue in the site of injury. The findings match patho-histological descriptions of the NF1 pseudarthrosis in the literature, where pseudarthrotic tissue is characterised as osteoid-rich, fibro-cartilaginous and highly vascularised tissue [7,8]. In the search for a possible therapeutic intervention as well as for the molecular mechanism of the disease, we tested the influence of statins on the process of bone repair in the Nf1Prx1 mouse model.

Statins are inhibitors of 3-hydroxy-3-methylglutaryl coenzyme A reductase, broadly used for the reduction of serum cholesterol. As statins inhibit the initial enzyme of the mevalonate pathway, they also reduce prenylation and farnesylation of signalling molecules, such as Ras and Ras-related proteins [9-11]. It has been well documented that statins induce a direct bone anabolic effect, which translates into accelerated bone healing in rats and mice [12-14]. In particular, simvastatin, mavastatin, fluvastatin and lovastatin have all been shown to stimulate bone formation [12]. Statin-induced bone formation is associated with increased osteoblast differentiation as measured by alkaline phosphatase, bone morphogenic protein 2 (BMP-2) and osteocalcin expression [15]. In addition, results of *in-vitro *experiments indicate that statins might inhibit bone resorption by interfering with osteoclast function in a similar way as bisphosphonates [16]. Both drug groups inhibit the mevalonate pathway albeit at different synthesis pathway levels, thus their mechanisms of action overlap. The clinical relevance of this remains unclear as several independent studies were published presenting contradictory results on the fracture risk reduction assessment in lovastatin-treated patients [17]. Independently of the bone anabolic and putative anti-catabolic properties, a potential usefulness of statins in the treatment of NF1 was suggested by the improvement of learning dysfunction in Nf1+/- mice [18]. Consequently, statins became our first choice for the treatment of the delayed bone injury repair in Nf1Prx1 mice. Here we present data showing that a high dose of systemically applied lovastatin improves bone repair in Nf1Prx1 mice. This is probably a result of normalisation of mitogen activated protein kinase (MAPK) signalling and enhanced Runx2 expression.

## Methods

### Animal procedures

The Nf1flox and Prx1Cre lines were maintained by continuous backcrossing to wild-type C57BL/6J mice to minimise genetic drift. The female Nf1flox mice were crossed to male Nf1flox heterozygous Prx1-Cre positive males. Mice were genotyped as described previously [19]. We used 12–14-week-old mice for cortical bone injury experiments, essentially as described in [5] with minor modifications. In brief, mice were anaesthetised by intraperitoneal injection of ketanest/rompun. The skin was shaved and skin incision made over the medial aspect of the proximal end of the tibia. Soft tissue was cleared away and a hole (500 μm diameter) through the tibia was made with a 0.5 mm stainless steel drill. The drill site was placed at the level of the distal end of the tibial crest through the entire diameter of the tibia, that is, through medial and lateral cortices and the intervening medulla. The skin was closed using acrylic histo-glue. Lovastatin was converted into its active sodium salt form as described previously [20]. In brief, 50 mg mevinolin in the lactone form (Sigma) was dissolved in 1 ml prewarmed (55°C) ethanol and 500 μl of 0.6 M NaOH was added. The solution was briefly vortexed and 10 ml of water was added. The solution was incubated for 30 minutes at room temperature. The final mevinolin solution (4 mg/ml) was adjusted to pH 8 with HCl and stored in multiple aliquots at -20°C. The treated group received daily oral gavage of 0.15 mg activated lovastatin in 150 μl end volume gavage. The same dose was shown to be effective in the treatment of the learning and attention deficits in the NF1 heterozygous knock-out mice [18]. All experimental procedures were approved by the Landesamt für Gesundheitsschutz und Technische Sicherheit (LaGeTSi), Berlin, Germany.

### Histological analysis

Tibiae were dissected with the surrounding soft tissue and fixed over night in phosphate buffered 4% paraformaldehyde (PFA). Subsequently tissue samples destined for calcified bone histology and micro computed tomography (μCT) analysis were processed according to the Technovit 9100 Kit manual (Heraeus Kulzer GmbH, Germany). Serial sections of 5 μm were cut with a hard tissue microtome and stained according to the VonKossa/Toluidine procedure. For paraffin embedding, tibiae were decalcified for 14 days while rotating at 4°C in phosphate buffered 4% PFA/0.5% ethylenediaminetetraacetic acid (EDTA) with one change of solution at day 7. Serial, 6 μm thick paraffin sections were prepared and used for Masson-Goldner staining, *in situ *hybridisation and tartrate-resistant acid phosphatase (TRAP) staining. TRAP histochemistry and TRAP-positive regions quantification was performed on the paraffin sections as described previously [4].

### *In situ *hybridisation

*In situ *hybridisations with Collagen1 and Osteopontin probes were performed using digoxigenin labelled cRNA probes as described previously [21]. The probes were amplified from the mouse embryonic day E17.5 cDNA library using the following primers:

Collagen1F: 5'-GGTACATCAGCCCGAACCCCAAGG-3'

Collagen1R: 5'-GTCTGGGGCACCAATGTCCAAGGG-3'

OsteopontinF: 5'-GATGAATCTGACGAATCTCAC-3'

OsteopontinR: 5'-CTGCTTAACCCTCACTAACAC-3'

The Runx2 expression was detected using ^32^P labelled cRNA probes as described previously [22]. The Runx2 probe was derived from mouse embryonic stage 14.5 cDNA library with the following primers:

Runx2F: 5'-GTGTTCTGTGGTCTCTGAG-3'

Runx2R: 5'-GGCAAAAGCTTGCAGAACTC-3'

Radioactive probe signals were photographed in dark field and the tissue histology was visualised using inverse phase optics.

### Three-dimensional imaging by μCT

Methacrylate embedded tibiae were scanned in plastic blocks using a vivaCT40 scanner from ScancoMedical. The following instrument settings were chosen for the measurements: voxel size 0.1 mm × 0.1 mm × 0.5 m; scan speed of 2 mm/second; contour mode 1; cortical threshold 350 mg/cm^3^. The cortical injury was located and a volume of interest (VOI) was defined comprising the complete injury site. To analyse bone formation within the bone marrow cavity, another VOI, comprising 90 consecutive scan slices, was selected reaching from the proximal to the distal callus end. All VOIs were analysed under identical settings with the Scanco evaluation software. Results of the μCT morphometric analysis were expressed as the mean ± standard deviation and statistical significance was examined using an unpaired *t*-test (*P < 0.05; **P < 0.01).

### Western blot

Calvarial bones (parietal and frontal bone) were harvested 7 days post injury. Bones were dissected free of connective tissue and muscles and homogenised in 300 μl radio immuno precipitation assay (RIPA) buffer supplemented with protease and phosphatase inhibitors using tissue homogeniser (Fisher Scentific). Homogenates were centrifuged for 5 minutes at 13000 rpm and the supernatants were collected. Whole-cell lysates of calvarial bones were resolved by electrophoresis in sodium dodecyl sulphate (SDS)-polyacrylamide gels and transferred onto polyvinylidenefluoride (PVDF) membranes (Amersham). For Western blot analysis, membranes were probed with the following antibodies: phospho-p42/44 (pERK1/2) #9102 Cell Signaling (diluted 1:1000), p44 (ERK1) #4372 Cell Signaling (diluted 1:1000).

### Serum deoxypyridinoline determination

Serum deoxypyridinoline (D-PYD) was measured with a METRA Serum PYD EIA Kit (Osteomedical GmbH) according to the supplied protocol.

## Results

**Figure 1:**
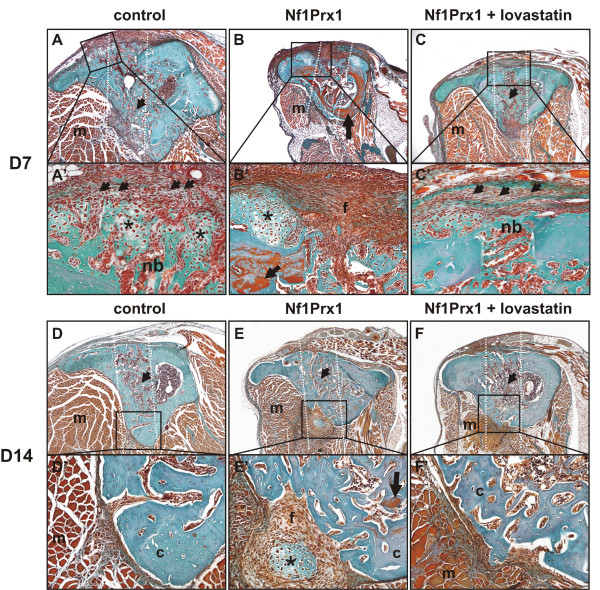
**Bone repair of the cortical tibia injury in Nf1Prx1 mice is accelerated by systemic high-dose lovastatin treatment**. (A)-(C') Masson-Goldner stained transverse paraffin sections of the drill channel (white dotted lines) 7 days post injury. (A), (A') New bone is formed in the marrow cavity in control animals (arrow). (A') The presence of cartilage suggests that cortical repair relies at least partially on endochondral bone formation (star). Recruited mesenchymal cells differentiate into osteoblasts embedded in the collagenous (green/blue) extracellular matrix (arrows). (B), (B') In mutant animals the entire cortical bone surrounding the injury site appears unmineralised as indicated by the orange stained matrix (arrow). Recruited fibroblasts fail to differentiate and collagenous matrix (see the green coloured matrix in (A')) is not produced. Formation of new bone in the bone marrow cavity is delayed, indicating a failure of repair process initiation. The cartilage is formed excessively (star) and the entire injury site is filled with fibro-cartilaginous tissue (f). (C), (C') Lovastatin treatment normalises the cortical bone quality around the injury site (note absence of orange staining). Recruited mesenchymal progenitor cells deposit green stained collagenous matrix (arrows). New bone is formed in the marrow cavity (arrow) as well as in the cortical region (nb). (D)-(F') Masson-Goldner staining of transverse sections of the injury site 14 days post induction. (D)-(E') Trabecular bone is present in the marrow space in control as well as in mutant animals (arrows). (E), (E') Nf1Prx1 mice exhibit persistence of fibrous (f) and cartilaginous (star) tissue in the area of the injury site. Reduced mineralisation is indicated by orange structures (arrow). (F), (F') No fibro-cartilaginous tissue is detected in the lovastatin-treated group. The cortical bone (c) in lovastatin-treated mice appears thicker and no signs of demineralisation are found; (m) denotes muscle.

**Figure 2:**
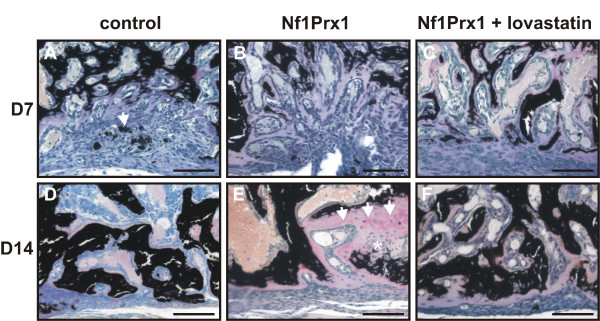
**Progression of cortical bone repair in control and Nf1Prx1 mice**. Toluidine/VonKossa stained transverse sections of the drill channel entry site 7 and 14 days post injury induction. (A), (B) Trabecular bone formation is delayed within the injury site in mutant mice as compared with controls at day 7. (C) Lovastatin treatment accelerates the formation of new mineralised bone by preventing fibro-cartilaginous tissue accumulation. (D), (E) Unlike in control mice, at day 14 post injury trabecular bone within the drill channel is covered by thick osteoid (arrows) in the mutant mice and the cartilaginous tissue persists (*). (F) Lovastatin treatment improves trabecular bone formation and extracellular matrix mineralisation. In addition, a marked reduction of the osteoid occurs.

**Figure 3:**
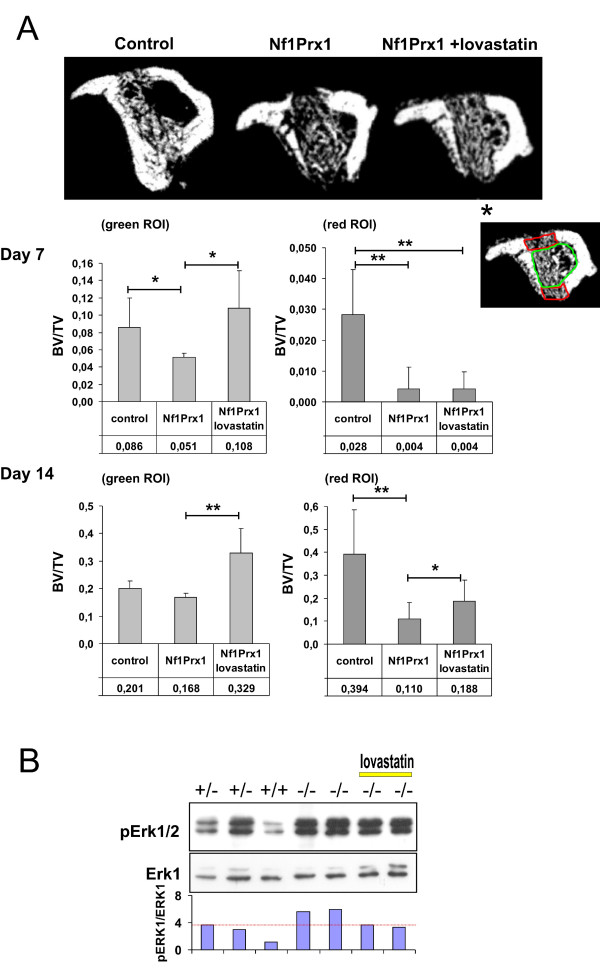
**Lovastatin treatment improves defect mineralisation in Nf1Prx1 mice**. (A) Transverse micro computed tomography sections of the injury 14 days post induction. Quantification of the mineralised matrix in the cortical regions (red region of interest (ROI)) and bone marrow shaft (green ROI) 7 days (*n *= 3) and 14 days (*n *= 6) post injury. Note the increased bone volume to total volume (BV/TV) in the bone marrow cavity (left chart) as well as in the cortical region (right chart) in animals treated for 7 and 14 days with lovastatin as compared with controls. (B) Analysis of the mitogen activated protein kinase (MAPK) pathway activation status (pErk1/Erk1 ratio) in calvaria bones of Nf1Prx1 mice and lovastatin-treated mice. The MAPK pathway activation was determined by densitometric analysis of the western blots.

The process of bone repair was examined 7, 14 and 28 days post injury in transverse serial sections of decalcified as well as calcified tibia (Figures 1 and 2). In addition, serial longitudinal sections were prepared in order to verify the location of the injury site and to follow bone healing in another plane (Additional file 1). The process of cortical and medullary defect healing was quantified with μCT at day 14 after injury (Figure 3).

### Day 7 post injury

**Figure 4:**
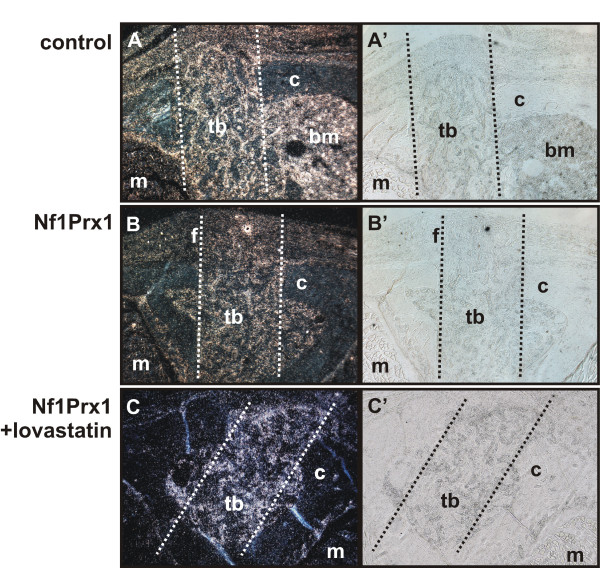
**Lovastatin treatment rescues Runx2 expression in the injury site**. (A)-(C) Runx2 expression analysed with radioactive *in situ *hybridisation on transverse sections of the drill site 7 days post injury. Representative sections of three tested animals are shown. (A')-(C') Tissue morphology visualised by bright field microscopy. (A) Intense Runx2 staining is detected throughout the bone marrow cavity (bm) and in the area occupied by newly forming trabecular bone (tb). (B) An overall decreased signal intensity in Nf1Prx1 mutants, with especially faint labelling in the area occupied by fibrous tissue (f). (C) Runx2 expression is restored within the trabecular bone formation area including the adjacent cortical defect region in lovastatin-treated mice.

In control mice the drill site was populated by connective tissue fibroblasts and mesenchymal progenitor cells at day 7 post injury. In agreement with data reported previously, new bone formation was initiated at the periosteal surface and in the bone marrow cavity (Figures 1A and 2A) [5]. The woven bone within the marrow cavity exhibited initial mineralisation as detected by VonKossa staining and small islands of mineralised tissue were detectable in the cortical region (white arrow in Figure 2A). Cartilage was also detected, indicating some degree of endochondral bone formation (Figure 1A and 1A'). In Nf1Prx1 mice connective tissue fibroblasts (f) were present in the injury site but the deposition of extracellular matrix associated with osteoblast differentiation did not occur (Figure 1B'). Consequently, the mineralisation process was delayed in the marrow cavity and only marginally present in the cortical region (Figure 2B and Additional file 1). Quantification by μCT showed that the bone volume to total volume fraction (BV/TV) in mutants was decreased by 40% in the bone marrow cavity (0.086 ± 0.034 versus 0.051 ± 0.005) and reduced five fold in the cortical regions (0.028 ± 0.015 versus 0.004 ± 0.007) when compared with controls (Figure 3A). Cartilage (*) was formed excessively and fibro-cartilaginous tissue accumulated on the margins (Figure 1B'). In addition, bone surrounding the drill site in Nf1Prx1 mice was generally unmineralised (Additional file 1 and Figure 1B). Unmineralised bone was detectable in all tested animals on the 7th and 14th day post injury (*n *= 9) but it was consistently absent in the non-injured Nf1Prx1 tibiae. Interestingly, this phenomenon was also observed at locations distant from the injury, suggesting the involvement of long-range and/or systemic signalling (longitudinal VonKossa/Toluidine stained sections, Additional file 1). *In situ *expression analysis showed a decreased level of Runx2 expression in the bone marrow cavity at day 7 post injury indicating impaired osteoblast formation and/or recruitment of progenitor cells (Figure 4B).

### Day 14 post injury

**Figure 5:**
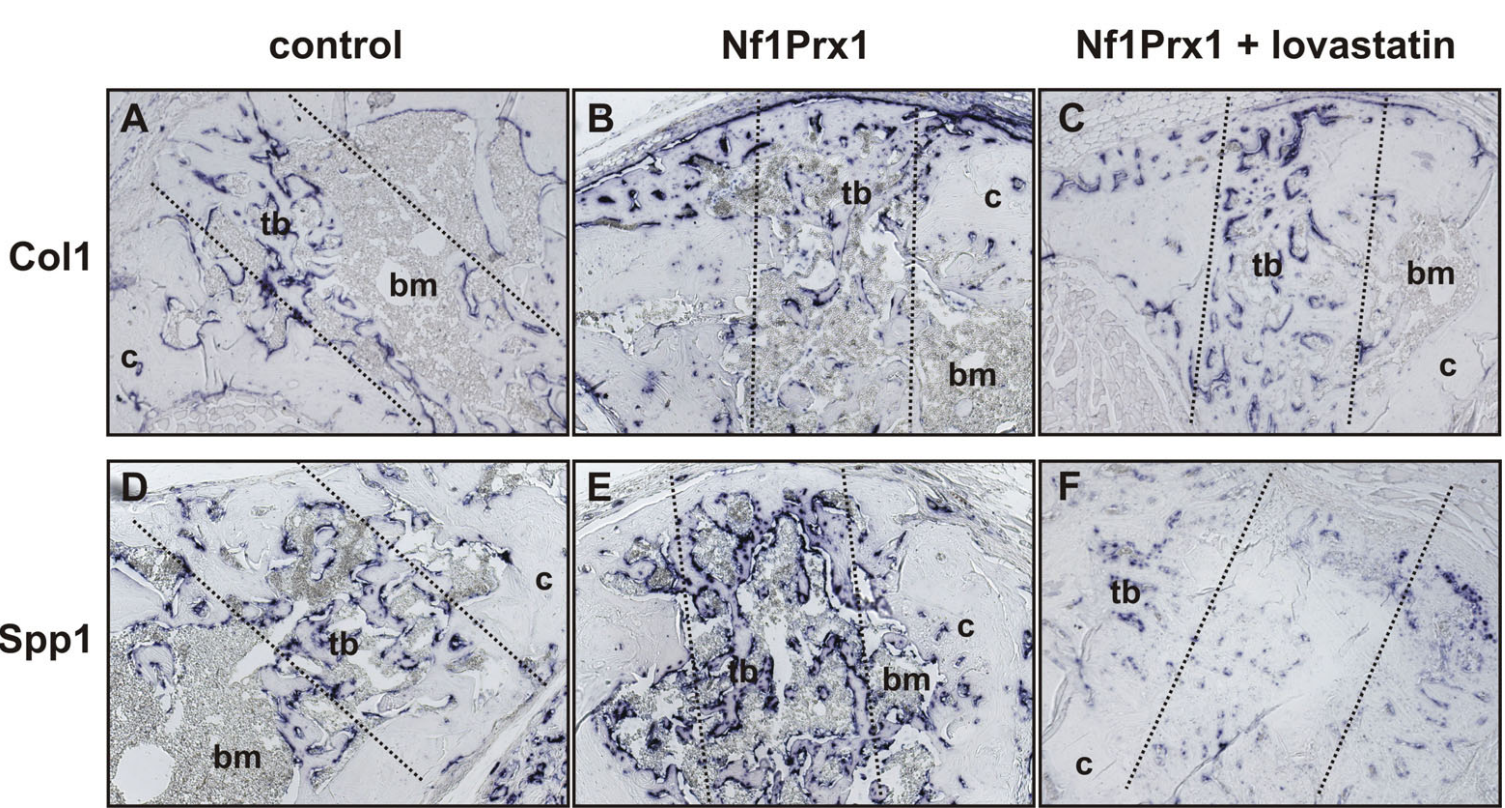
**Lovastatin treatment reduces Osteopontin expression within the injury site in the Nf1Prx1 mice**. (A)-(C) Collagen1 and (D)-(F) Osteopontin expression visualised by *in situ *hybridisation in the drill channel (black dotted lines) 14 days post injury induction. Representative sections of three animals are shown. (A)-(C) Newly formed trabecular bone (tb) within bone marrow cavity (bm) is lined with collagen1 positive osteoblasts (blue staining). (D)-(E) Osteopontin positive cells appear more frequent and the expression level is higher in mutant mice as compared with controls. (F) Lovastatin treatment, while increasing the trabecularisation (see Figure 3 for quantification of bone volume), reduces Osteopontin expression. (c) Cortical bone.

At day 14 post injury the drill site in control animals was filled with newly formed bone, osteoid and a few blood vessels (Figures 1D, 1D' and 2D). Mineralisation islands had developed to trabeculae by replacing cartilage and fibrous tissue. The newly formed trabeculae were thicker, and lined with a thin osteoid indicating timely mineralisation of the newly formed matrix (Figure 2D). In the mutant mice lamellar bone was formed within the bone marrow cavity and in the cortical defect. However, its mineralisation was retarded, as most trabeculae remained covered with a thick layer of osteoid (Figure 2E). In addition, trabeculae within the cortical regions appeared scarcer and the cartilaginous and fibrotic tissues persisted (Figure 1E and 1E'). Quantitative μCT analysis revealed a 75% reduction of BV/TV in the cortical regions (0.394 ± 0.19 versus 0.11 ± 0.07) as well as slight decrease in the average BV/TV in the bone marrow cavity (0.201 ± 0.26 versus 0.168 ± 0.16) when compared with the control group (Figure 3A). This appears not to be due to a scarcity of osteoblasts, as judged by the presence of the collagen type I expressing cells (Figure 5B). The intense expression of Osteopontin, known to demarcate terminally hypertrophic chondrocytes and early osteoblasts, argues for an impairment of the maturation process (Figure 5E). Osteopontin is known to facilitate osteoclast mediated bone resorption [23,24]. We thus quantified TRAP-positive bone lining cells within the injury site and determined the rate of bone turnover by measuring D-PYD concentrations in serum. Osteoclast numbers were increased in the injury site in Nf1Prx1 mice when compared with controls and lovastatin treatment did not significantly change this (data not shown). This was paralleled by an increased serum D-PYD in Nf1Prx1 animals, which was only slightly reduced by lovastatin treatment (control 2.5 ± 0.39 nMol/l; Nf1Prx1 3.95 ± 0.71 nMol/l; Nf1Prx1 +lovastatin 3.49 ± 0.83; *n *= 3).

### Day 28 post injury

The injury site became difficult to locate in the control animals, demonstrating the speed and efficacy of the regeneration processes. Solid bone, undistinguishable from the surrounding cortical bone, replaced initially formed woven bone. In the Nf1Prx1 animals the injury site was also closed by calcified extracellular matrix, but woven bone was still present in the marrow cavity. Furthermore, cortical bone was covered by a thick osteoid, indicating an ongoing abnormality of the mineralisation process. Cortical bone appeared strikingly thinned and at many sites it was penetrated by thick blood vessels (Additional file 2).

### Lovastatin treatment improves injury healing in Nf1Prx1 mice

#### Day 7 post injury

Improvement of bone quality had already become obvious by the 7th day of treatment. In contrast to untreated mice, unmineralised bone was neither detectable in the vicinity nor distally from the injury site (Figures 1C and 2C). Calcified trabecular bone was found in the bone marrow cavity and it became detectable also in the cortical regions, indicating accelerated osteoprogenitor differentiation as well as normalisation of mature osteoblast function. The μCT analysis indicated that BV/TV within the bone marrow cavity was two-fold higher in the lovastatin-treated mice than in untreated mice (0.108 ± 0.043 versus 0.051 ± 0.005) and slightly exceeded the BV/TV values of the control group (0.086 ± 0.034) (Figure 3A). In contrast, in the cortical regions BV/TV remained at basal level in both the lovastatin-treated and untreated Nf1 deficient mice compared with the control group (0.004 ± 0.007 and 0.004 ± 0.0050 versus 0.028 ± 0.015, respectively). Interestingly, cartilage present in the control animals and excessively formed in the untreated mutants was absent suggesting that desmal ossification is promoted by the lovastatin treatment (Figure 1C and 1C'). This was further corroborated by *in situ *analysis, showing an increased expression of Runx2 on the 7th day post injury in the marrow cavity (Figure 4C).

#### Day 14 post injury

On day 14 post injury a marked increase of BV/TV was detected within the bone marrow cavity of lovastatin treated mice as compared with untreated mutants (0.168 ± 0.16 versus 0.329 ± 0.089; Figure 3A). A less pronounced BV/TV increase was also detectable in the cortical regions (0.110 ± 0.071 versus 0.188 ± 0.092; Figure 3A). Thus, lovastatin appears to accelerate cortical bone repair primarily by enhancing new bone formation within the bone marrow cavity and by replacing fibro-cartilaginous tissue in the injury site with mineralised bone matrix (Figure 1F and 1F'). The associated trabecullar bone lining osteoblasts expressed Collagen type I and little Osteopontin, which is characteristic of the mature osteoblast phenotype (Figure 5C and 5F). The osteoid thickening characteristic for Nf1Prx1 mice was no longer observed, indicating that osteoblast function was restored. Consistent with the function of lovastatin as an indirect inhibitor of Ras prenylation, the bone pro-anabolic effect of lovastatin correlated with the normalisation of MAPK signalling measured as a phospho-Erk1/2 to Erk1 ratio in calvarial osteoblasts (Figure 3B).

## Discussion

Studies of Nf1 function in bone development and homeostasis have long been hampered by the lack of a suitable animal model. Recently, we have shown that bi-allelic inactivation of the Nf1 gene in developing limbs leads to a phenotype which recapitulates features of NF1-associated bone dysplasia, including bowing of the tibia. Despite a striking decrease of the bone mineral content and increased bone porosity Nf1 inactivation does not result in spontaneous fractures [4]. We therefore decided to induce a bone injury in the Nf1-deficient limb in order to model aspects of NF1-associated tibial fractures and pseudarthrosis. The cortical bone injury model presented here uncovers an important role for Nf1 in the regulation of bone regeneration. The study by Yu and colleagues conducted on Nf1 heterozygous mice revealed no dramatic changes in bone morphometric parameters and dynamics of bone formation [25]. These results are in agreement with ours, as heterozygous Nf1flox Prx1Cre mice did not differ in the progression of injury repair from wild-type mice (data not shown). In contrast, Nf1 deficiency results in delayed osteoblast differentiation, leading to a retardation of the repair process. This is paralleled by ectopic cartilage formation as well as an expansion of spindle-shaped connective tissue fibroblasts, both also found in the NF1 pseudarthrosis tissue [7]. In addition, our data show an increased number of osteoclasts at the injury site (data not shown) paralleled by an increased serum D-PYD concentration in the mutant animals. Both effects are only marginally reduced by lovastatin treatment. The increase of osteoclast number in the injury site is similar to our previous finding of the increased osteoclast number in the chondro-osseous junction [4]. This effect is likely cell non-autonomous, as Nf1 is not inactivated in Nf1Prx1 osteoclasts.

The injury results in the occurrence of unmineralised bone, which is present not only in the vicinity of the drill site, but also at sites distant to it. The aetiology remains obscure but the phenomenon seems to be important for understanding the nature of NF1 pseudarthrosis. We hypothesise that the injury-induced demineralisation process is driven by locally and systemically secreted factors. NF1 is associated with decreased bone mineral content and in acute cases osteomalacia/rickets of unknown aetiology has been observed [26-28]. A tumour inductive role has also been suggested [29]. Our results indicate that in Nf1 deficient limbs, the injury itself triggers a partial demineralisation of the neighbouring bone. The mechanism behind this process as well as the nature of the involved signalling pathways awaits future investigation.

Statins have been shown to promote fracture healing in wild-type mice and rats [12-14]. Inhibition of the mevanolate pathway and the BMP2-dependent bone anabolic action of lovastatin are likely to be involved [30]. In the context of Nf1 deficiency lovastatin's activity as a posttranslational inhibitor of Ras seems to be of central importance [31]. We and others have shown that de-repression of MAPK pathway signalling in the absence of Nf1 hinders osteoblastic differentiation and prevents extracellular matrix mineralisation [4,32]. Complementarily, a recent report by Kono and colleagues indicates that MAPK pathway inhibition promotes matrix mineralisation [33]. In this context our current data argues for a bone anabolic action of statins being at least partially dependant on the inhibition of the Ras/MAPK pathway. Further studies are necessary to determine the exact mechanism, but the principle of MAPK involvement in osteoblastogenesis emerges and statins seem an attractive pharmacological tool for modulating this crucial signalling pathway. Interestingly, local statin delivery in the fracture site was recently shown to accelerate bone healing in mice and rats [12,13]. In summary, our results confirm the validity of the hypothesis that statins have a beneficial influence on the defective bone healing in Nf1 deficiency. They also set the stage for future experiments aimed at the treatment of the focal NF1 bone changes with local statin delivery. The presented mouse model recapitulates multiple aspects of NF1 pseudarthrosis and can be envisioned as an important tool facilitating pre-clinical stage testing of other drugs targeting NF1-related skeletal abnormalities.

## Abbreviations

μCT: microcomputed tomography; BMP-2: bone morphogenic protein 2; BV/TV: bone volume/total volume; D-PYD: deoxypyridinoline; EDTA: ethylenediaminetetraacetic acid; MAPK: mitogen activated protein kinase; Nf1: neurofibromin; NF1: neurofibromatosis type 1; Nf1Prx1: Nf1flox × Prx1Cre positive; PFA: paraformaldehyde; PVDF: polyvinylidenefluoride; RIPA: radio immuno precipitation assay; SDS: sodium dodecyl sulphate; TRAP: tartrate-resistant acid phosphatase; VOI: volume of interest.

## Competing interests

The authors declare that they have no competing interests.

## Authors' contributions

MK, SM conceived and coordinated the study and drafted the manuscript and MK also performed the surgical procedures, paraffin and methacrylate histology and Western blot analysis. BT and NK were involved in the execution of animal work, and the *in situ *analysis. JK performed and evaluated μCT analysis, analysed the methacrylate histology and helped to prepare the manuscript. MO and SS performed tissue processing, mouse work and genotyping. UK reviewed the manuscript.

## Pre-publication history

The pre-publication history for this paper can be accessed here:



## Supplementary Material

Additional file 1 **Progression of bone repair in control and Nf1Prx1 mice, longitudinal view**. Toluidine/VonKossa stained longitudinal methacrylate sections of wild-type and Nf1Prx1 tibia 7 and 14 days post injury induction. Uninjured tibia is shown for comparison. The trabecular bone formed within the bone marrow cavity demarcates the injury site. Magnification of the cortical bone distant from the injury site shows normal mineralisation in uninjured animals and partial cortical bone demineralisation in mutant mice 7 and 14 days post injury (red frame, arrows). Click here for file

Additional file 2 **Progression of bone repair in control and Nf1Prx1 mice, 28 days post injury**. Toluidine/VonKossa stained transverse sections of the cortical defect area. After 28 days post injury the cortical structure is regenerated in control mice (left). The cortical bone in mutant mice remains thinned and overlaid by a thick osteoid (red arrows). It is also excessively penetrated by blood vessels (white arrows). Click here for file
