# Supplementary figures and images for: Modelling neurofibromatosis type 1 tibial dysplasia and its treatment with lovastatin
Source: BMC Med. 2008 Jul 31;6:21. doi: 10.1186/1741-7015-6-21 (PMC2516519; doi:10.1186/1741-7015-6-21)

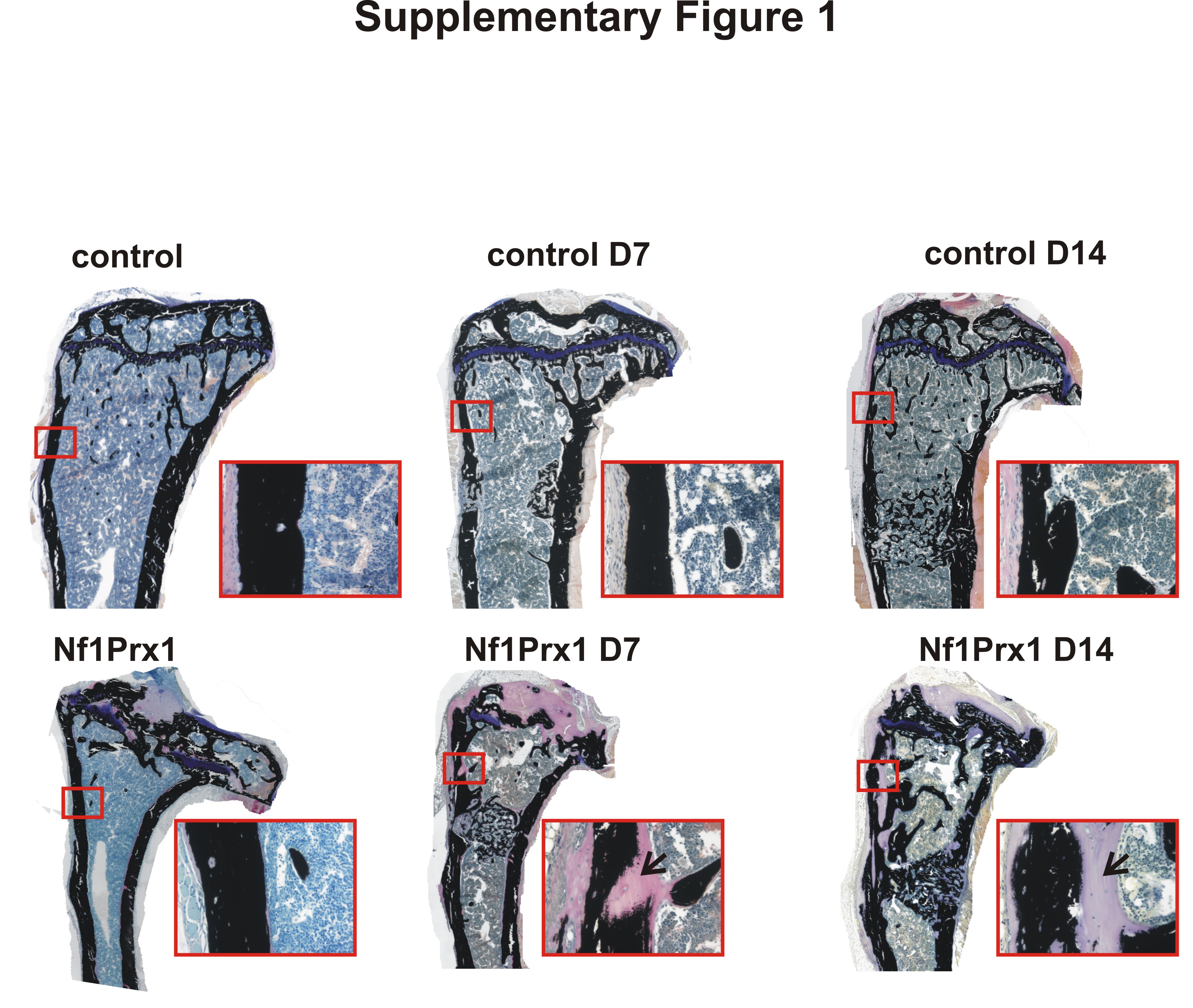

Supplement: Additional file 1 — Progression of bone repair in control and Nf1Prx1 mice, longitudinal view. Toluidine/VonKossa stained longitudinal methacrylate sections of wild-type and Nf1Prx1 tibia 7 and 14 days post injury induction. Uninjured tibia is shown for comparison. The trabecular bone formed within the bone marrow cavity demarcates the injury site. Magnification of the cortical bone distant from the injury site shows normal mineralisation in uninjured animals and partial cortical bone demineralisation in mutant mice 7 and 14 days post injury (red frame, arrows). [file 1741-7015-6-21-S1.jpeg]

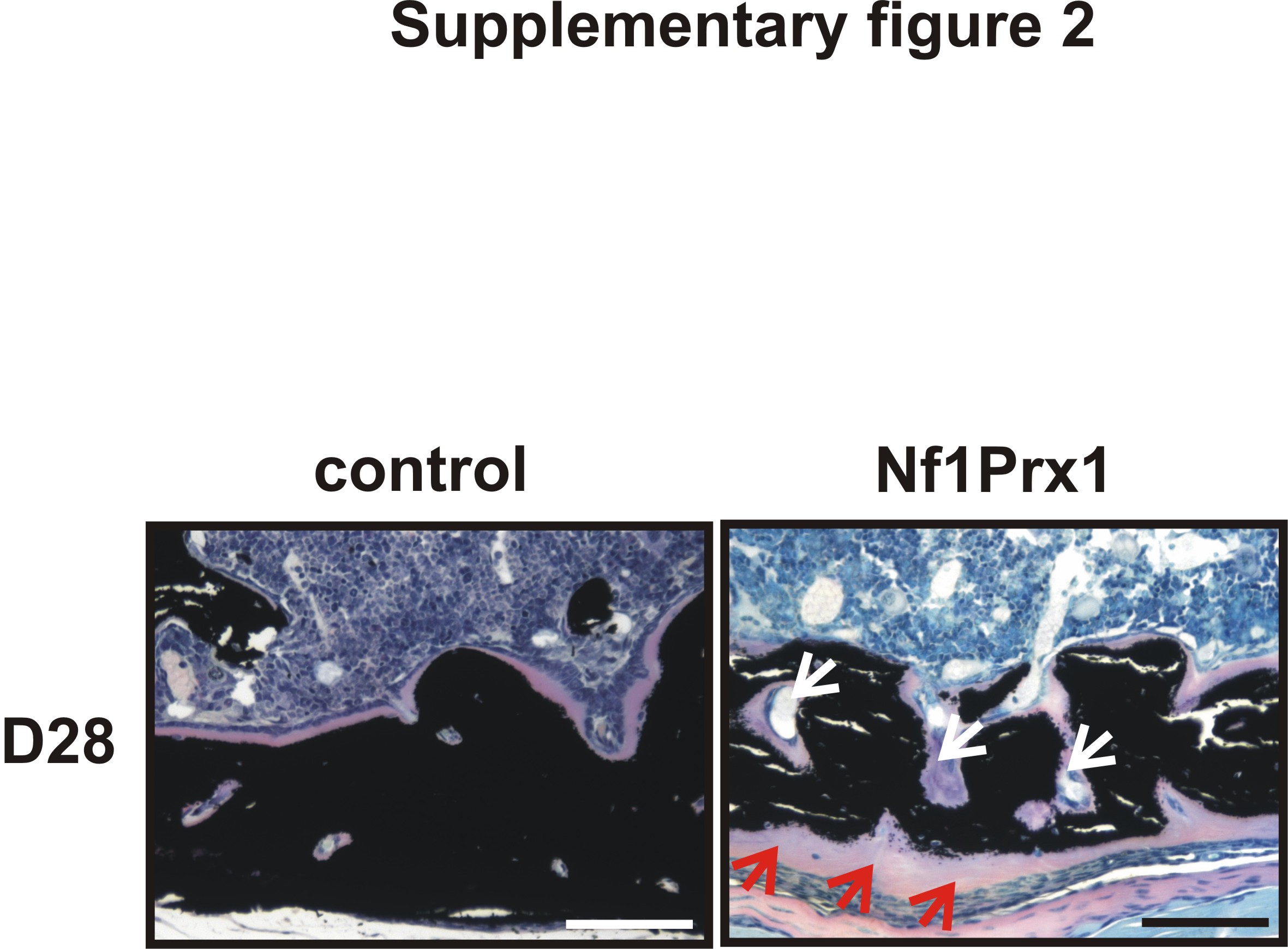

Supplement: Additional file 2 — Progression of bone repair in control and Nf1Prx1 mice, 28 days post injury. Toluidine/VonKossa stained transverse sections of the cortical defect area. After 28 days post injury the cortical structure is regenerated in control mice (left). The cortical bone in mutant mice remains thinned and overlaid by a thick osteoid (red arrows). It is also excessively penetrated by blood vessels (white arrows). [file 1741-7015-6-21-S2.jpeg]
